# The Gαs‐protein‐mediated pathway may be steadily stimulated by prostanoid EP2 receptors, but not by EP4 receptors

**DOI:** 10.1002/2211-5463.13378

**Published:** 2022-02-15

**Authors:** Keijo Fukushima, Kanaho Senoo, Naoki Kurata, John W. Regan, Hiromichi Fujino

**Affiliations:** ^1^ Department of Pharmacology for Life Sciences Graduate School of Pharmaceutical Sciences & Graduate School of Biomedical Sciences Tokushima University Japan; ^2^ Laboratory of Chemical Pharmacology Graduate School of Pharmaceutical Sciences Chiba University Japan; ^3^ Department of Pharmacology & Toxicology College of Pharmacy The University of Arizona AZ USA

**Keywords:** biased signaling, black/Leff operational model calculation, cAMP, PDE, prostanoid EP2 receptor, prostanoid EP4 receptor

## Abstract

EP2 and EP4 prostanoid receptors have long been considered to have similar roles, since they are known to couple with Gαs‐protein and activate cAMP‐mediated signaling pathways. In this study, we re‐evaluated the results of cAMP assays with or without phosphodiesterase (PDE) inhibitor pretreatment. Here, we show that in the absence of PDE inhibitor pretreatment, prostaglandin E_2_ causes accumulation of cAMP in EP2 receptors, whereas markedly low levels of cAMP accumulated in EP4 receptors. By applying the Black/Leff operational model calculation, we found that EP2 receptors have a biased ability to intrinsically activate the Gαs‐protein‐mediated pathway, whereas EP4 receptors have strong biased activity for the Gαi‐protein‐mediated pathway. Thus, EP2 and EP4 receptors may not be similar Gαs‐coupled receptors but instead substantially different receptors with distinct roles.

## Graphical abstract

EP2 and EP4 receptors have long been considered to share their roles and signaling; however, they may be substantially different receptors with distinct roles.

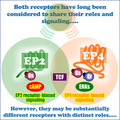

**Abbreviations**
cAMP: cyclic AMP
DMEM: Dulbecco's modified Eagle's medium
EP: E‐type prostanoid
ERKs: extracellular signal‐regulated kinases
HEK‐EP2 cells: HEK‐293 cells stably expressing human EP2 receptors
HEK‐EP4 cells: HEK‐293 cells stably expressing human EP4 receptors
IBMX: isobutylmethylxanthine
PDE: phosphodiesterase
PGE_2_
: prostaglandin E_2_
PI3K: phosphatidylinositol 3‐kinase
PKA: protein kinase A
TCF: T‐cell factor

The physiological effects of prostaglandin E_2_ (PGE_2_) are largely mediated by four primarily E‐type prostanoid (EP) receptor subtypes known as EP1, EP2, EP3, and EP4 [1, 2]. Among the four subtypes, EP2 and EP4 receptors are both known to couple with Gαs‐protein. However, before the molecular cloning of these receptors, the Gαs‐coupled and cyclic AMP (cAMP)‐producing EP receptor subtypes were thought to be one single subtype, which had been defined pharmacologically as the EP2 receptor subtype [2]. In 1993, the first cAMP‐producing EP receptors were cloned in mouse and humans, and they were named EP2 receptors [3]. A year later, the newly cloned second cAMP‐producing human EP receptors, i.e., the fourth EP receptors, were found [4], leading to confusion in 1994 [2, 5]. Indeed, the newly cloned receptors were sensitive to the pharmacological EP2 receptor agonist, butaprost, whereas the first receptors were not [4]. Therefore, the butaprost‐sensitive, the new, and the fourth EP receptors were designated as EP2 receptors, whereas the prior‐cloned butaprost‐insensitive EP2 receptors then renamed EP4 receptors [1, 2, 4, 6]. Although many studies have been reported for more than a quarter of a century, decisive evidence of the significant differences has not been established in terms of the second messenger signaling of these two receptors.

However, the differences between the two receptors have been begun to be reported. Regarding agonist‐induced desensitization and internalization of both receptors [7, 8], the EP4 receptors, but not EP2 receptors, were reported to undergo PGE_2_‐induced desensitization [7] and internalization [8]. Thereafter, in 2005, additional Gαi‐protein coupling was found in EP4 receptors, but not in EP2 receptors [9], which opened the discussion about the possibility that EP4 receptors function in multiple signaling pathways and have biased activities. The additional Gαi‐protein coupling with the EP4 receptors may be the reason why EP4 receptors had lower cAMP production [5, 9] and weaker protein kinase A (PKA) activity [5, 10] than EP2 receptors.

Recently, 15‐keto‐PGE_2_, a metabolite of PGE_2_, was reported to act as a switch for cellular signaling to the EP2 receptor‐mediated pathway from the EP4 receptor‐mediated pathway [11]. Thus, PGE_2_‐initiated EP4 receptor‐mediated signaling may be terminated by the subsequent 15‐keto‐PGE_2_‐adopted EP2 receptor‐mediated signaling if both receptors are expressed on nearby tissues/cells.

These studies suggested that 15‐keto‐PGE_2_ is not a nonfunctional metabolite of PGE_2_, and that EP2 and EP4 receptors may share roles in inflammatory responses; PGE_2_‐stimulated EP4 receptors pass on activities to EP2 receptors, which are activated by 15‐keto‐PGE_2_ as a switching agonist [11]. As PGE_2_ is well known to play a role in inflammation, the novel role‐sharing mechanisms regulated by EP2 and EP4 receptors may be significant for terminating PGE_2_‐evoked inflammation and/or maintaining the homeostasis, e.g., of colorectal tissues/cell functions.

However, these previous discussions/suggestions were based on the estimated maximal cAMP formed by receptor activation by prostanoids, but the formed cAMP is degraded following the activation of phosphodiesterase (PDE) to some extent. Thus, the practical amounts of cAMP and cAMP‐mediated signaling may be smaller and weaker than previously reported. To improve the estimation, we re‐calculated and re‐evaluated the simulation using cAMP assays stimulated by either EP2 receptors or EP4 receptors with PGE_2_, which were performed with or without PDE inhibitor pretreatment.

Without PDE inhibitor pretreatment, PGE_2_ led to the accumulation of cAMP in EP2 receptors. In EP4 receptors, however, PGE_2_ led to the accumulation of markedly low levels of formed cAMP, without PDE inhibitor pretreatment. Although it has been reported that the *E*_max_ level of PGE_2_‐stimulated cAMP formation in HEK‐293 cells stably expressing human EP4 receptors (HEK‐EP4 cells) is lower than that in HEK‐293 cells stably expressing human EP2 receptors (HEK‐EP2 cells) [5, 9, 10], the practical amounts of the accumulated cAMP under physiological conditions in HEK‐EP4 cells may be much lower than previously considered. This strongly suggested that EP2 receptors steadily stimulate the Gαs‐protein‐mediated pathway, whereas EP4 receptors are unlikely to primarily activate the Gαs‐protein‐mediated pathway and instead activate the Gαi‐protein‐mediated pathway. We here propose that EP2 and EP4 receptors are not role‐sharing complementary receptors, but substantially different receptors, with distinct roles in maintaining homeostasis in a coordinated manner.

## Materials and methods

### Cell culture and materials

HEK‐EP2 cells or HEK‐EP4 cells [11] were cultured in Dulbecco’s modified Eagle’s medium (DMEM; Nacalai Tesque, Kyoto, Japan) containing 10% FBS (Thermo Fisher Scientific, Waltham, MA, USA), 250 μg·mL^−1^ of geneticin (Phyto Technology Laboratories, Shawnee Mission, KA, USA), 200 μg·mL^−1^ of hygromycin B (Enzo Life Science, Farmingdale, NY, USA), and 100 μg·mL^−1^ of gentamicin (Life Technologies, Carlsbad, CA, USA) at 37 °C. PGE_2_ was obtained from Cayman Chemical (Ann Arbor, MI, USA). All materials were purchased from Wako Pure Chemical (Osaka, Japan) unless otherwise stated.

### cAMP assay

HEK‐EP2 cells or HEK‐EP4 cells were cultured in 6‐well plates and switched 16 h prior to the experiments from DMEM to Opti‐MEM (Thermo Fisher Scientific) containing 250 μg·mL^−1^ of geneticin, 200 μg·mL^−1^ of hygromycin B, and 100 μg·mL^−1^ of gentamicin at 37 °C. Approximately, 5 × 10^5^ cells per well were treated either with or without 0.1 mg·mL^−1^ of isobutylmethylxanthine (IBMX; Sigma‐Aldrich, St. Louis, MO, USA) for 25 min, followed by treatment with vehicle (0.1% Me_2_SO), or with the indicated concentrations of PGE_2_ for the indicated times at 37 °C. The amount of cAMP formed was calculated from a standard curve prepared using non‐radiolabeled cAMP, as described previously [11].

### Black/Leff operational model calculation

**Fig. 1:**
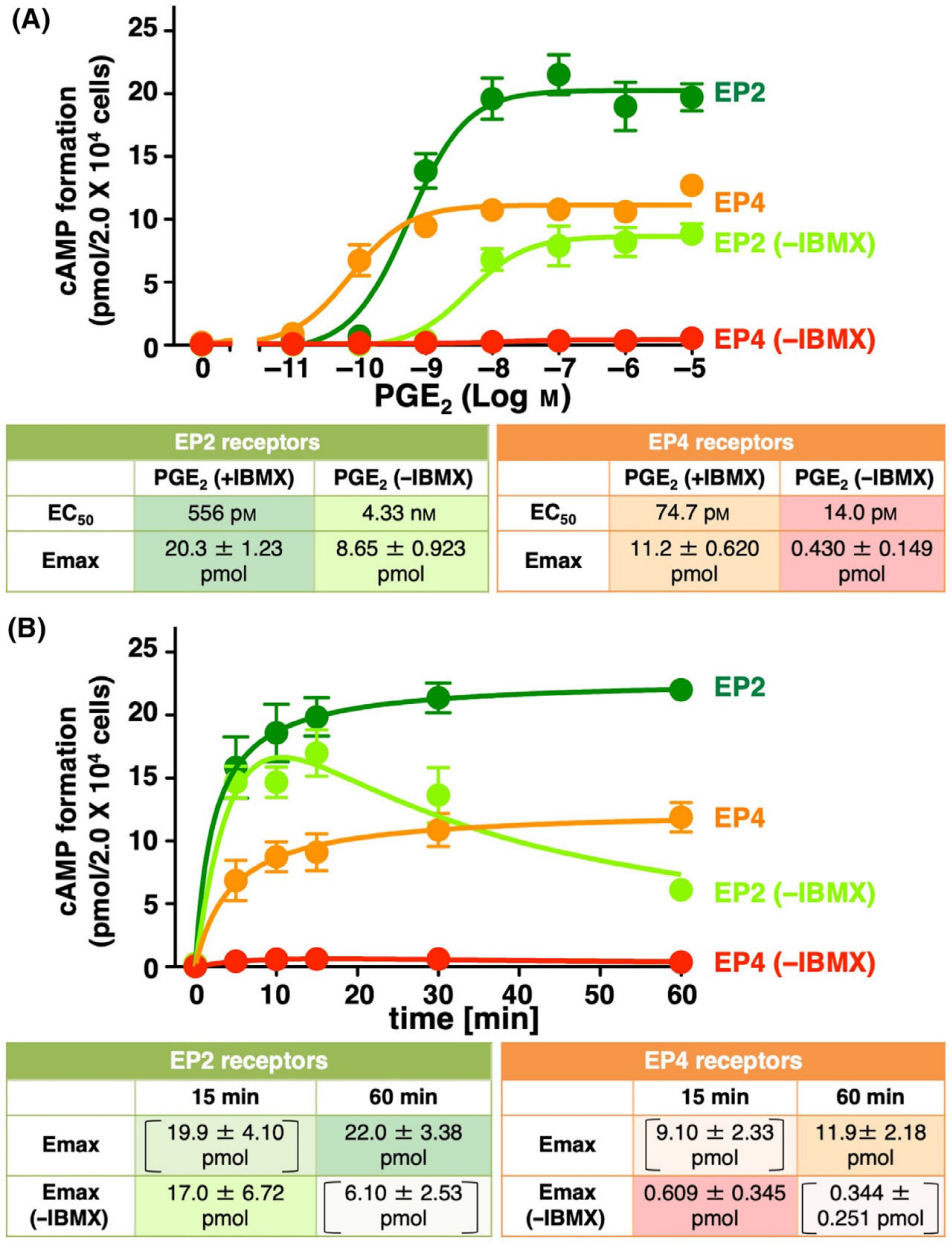
Effects of PGE_2_ with or without IBMX pretreatment on cAMP formation in HEK‐EP2 and HEK‐EP4 cells. (A) HEK‐EP2 cells or HEK‐EP4 cells were pretreated with or without IBMX and then treated with vehicle or the indicated concentration of PGE_2_ for 60 min in the cAMP assay. The tables show EC_50_ values and *E*_max_ values of PGE_2_‐stimulated formation of cAMP with or without IBMX in HEK‐EP2 cells or HEK‐EP4 cells. (B) HEK‐EP2 cells or HEK‐EP4 cells were cultured, pretreated with or without 0.1 mg·mL^−1^ of IBMX for 25 min, and then stimulated with 10‐nm PGE_2_ (HEK‐EP2 cells) or 100‐nm PGE_2_ (HEK‐EP4 cells) for the indicated time. The green line: PGE_2_‐stimulated cAMP formed from EP2 receptors under the influence of IBMX, orange line: PGE_2_‐stimulated cAMP formed from EP4 receptors under the influence of IBMX, lime green line: PGE_2_‐stimulated cAMP formed from EP2 receptors without IBMX pretreatment, orange‐red line: PGE_2_‐stimulated cAMP formed from EP4 receptors without IBMX pretreatment. The tables show EC_50_ and *E*_max_ values of PGE_2_‐stimulated formation of cAMP with or without IBMX in HEK‐EP2 cells or HEK‐EP4 cells. The numbers in the brackets are the amounts of cAMP formed at the indicated time points, which are apparent/pseudo *E*_max_ values needed for calculations. The amounts of cAMP formed are shown in pmol/2.0 × 10^4^ cells/sample and are the mean ± SEM of at least three independent experiments, each performed in duplicate. The amounts of cAMP formed are shown in pmol/2.0 × 10^4^ cells/sample and are the mean ± SEM of at least three independent experiments, each performed in duplicate.

The apparent affinity (*K*_A_) and Tau value of PGE_2_ without IBMX were determined using graphpad prism software (version 8.0.1, La Jolla, CA, USA) as the hypothetical partial agonist relative to the value with IBMX as the full agonist. The equation “Operational model‐Partial agonist” was applied using the value of EC_50_ from Fig. 1A and the value of cAMP formation at each time point (15 min and 60 min) in Fig. 1B as each *E*_max_ using the formulas below. The basal level was 0, and Hill slopes used were specified as 1. The *E*_max_ value with IBMX was used as the Effect_max_.

$$\mathrm{Operate}=\frac{10^{\log K_{A}}+10^{\left[A\right]}}{10^{\mathrm{logTau}+[A]}}$$

$$Y=\mathrm{Basal}+\;\frac{{\mathrm{Effect}}_{\max}‐\mathrm{Basal}}{1+10^{\mathrm{Operate}}}$$

[*A*]: PGE_2_ concentration; *Y*: cAMP formation.

In cAMP formation, for hypothetical partial agonists (without IBMX), the transduction coefficient (log *R*, *R* = (Tau/*K*_A_)) was obtained from *K*_A_ and Tau calculated by the Black/Leff operational model. For full agonists (with IBMX), log *R* was directly calculated by the formula below. The basal level was 0, and Hill slopes used were specified as 1. The *E*_max_ value with the IBMX obtained at each time point [12] was used as the Effect_max_. The log *R* of other pathway [extracellular signal‐regulated kinases (ERKs)‐mediated signaling and β‐catenin/T‐cell factor (TCF)‐mediated signaling] was calculated from the values of EC_50_ and *E*_max_ in the previous report [11]. Briefly, the EC_50_ values and *E*_max_ values of ERKs‐mediated signaling in EP2 receptors were 12.6 nm and 7.65, whereas in EP4 receptors were 0.863 nm and 53.3, respectively [11]. In the case of β‐catenin/TCF‐mediated signaling, the EC_50_ values and *E*_max_ values in EP2 receptors were 0.123 nm and 454, whereas in EP4 receptors were 0.0654 nm and 471, respectively [11].

$$Y=\mathrm{Basal}+\frac{{\mathrm{Effect}}_{\max}‐\mathrm{Basal}}{1+\frac{1+\frac{\left[A\right]}{10^{\mathrm{Log}K_{A}}}}{\left[A\right]\times 10^{\mathrm{Log}(\mathrm{Tau}/K_{A})}}}$$

[*A*]: PGE_2_ concentration; *Y*: cAMP formation.

## Results and Discussion

Practically, under physiological conditions, the formed cAMP is degraded following the activation of PDE to some extent, and the amount of cAMP and cAMP‐mediated signaling will be smaller and weaker than previously reported. Thus, we previously demonstrated that the poor survival rate of colorectal cancer may be related to the lower activation of cAMP‐mediated signaling ascribed to the lower expression level of EP2 receptors in the tissue [11] because cAMP‐mediated signaling is widely accepted to regulate the inhibition of cellular growth [13]. We also previously reported that PGE_2_‐initiated EP4 receptor‐mediated cellular growth signaling is terminated by the subsequent 15‐keto‐PGE_2_‐adopted EP2 receptor‐mediated cell growth inhibition signaling required for maintaining homeostasis [11]. Hence, we discussed the importance of continuous cAMP‐mediated signaling for maintaining homeostasis via 15‐keto‐PGE_2_‐activated EP2 receptors. However, we have been performed the cAMP assay under the influence of the PDE inhibitor IBMX. Therefore, what we have discussed previously were estimated based on the potential maximal cAMP formed through the EP2 and/or EP4 receptors activation by their ligands, e.g., PGE_2_.

To improve our estimation, we re‐calculated and re‐evaluated the simulation using cAMP assays performed with or without IBMX.

As shown in Fig. 1A and as reported previously [11], when cells were treated for 60 min with indicated concentrations of PGE_2_, the EC_50_ value in HEK‐EP2 cells was 556 pm (95% confidence interval: 326–948 pm), and the *E*_max_ value was 20.3 ± 1.23 pmol when IBMX was pretreated, *i.e*., under the influence of a PDE inhibitor. When cells were not pretreated with IBMX, the EC_50_ value in HEK‐EP2 cells were shifted to the right; 4.33 nm (95% confidence interval: 1.75–10.7 nm), and the *E*_max_ value decreased by more than half (8.65 ± 0.923 pmol).

In the case of EP4 receptors, when cells were pretreated with IBMX and then treated for 60 min with indicated concentrations of PGE_2_, the EC_50_ value in HEK‐EP4 cells was 74.7 pm (95% confidence interval: 40.3–138 pm), and the *E*_max_ value was 11.2 ± 0.62 pmol, similar to that previously reported [11]. Of note, when cells were not pretreated with IBMX, PGE_2_ formed cAMP at any concentration examined; thus, the calculated EC_50_ values and *E*_max_ values were scattered and likely inaccurate; EC_50_ value was 14.0 nm (95% confidence interval: 512 pm–384 nm); and the *E*_max_ value was 0.430 ± 0.149 pmol. Based on Fig. 1A, the practical amounts of cAMP and cAMP‐mediated signaling are smaller and weaker than those previously reported.

As shown in Fig. 1A, the practical amounts of cAMP formed by the activation of the EP4 receptors remain unclear, at least at 60 min after the stimulation with PGE_2_. However, without IBMX pretreatment, it is possible that the formed cAMP is degraded by PDE following the activation of adenylyl cyclase. Therefore, HEK‐EP2 cells were treated with 10‐nm PGE_2_, and HEK‐EP4 cells were treated with 100‐nm PGE_2_ for the indicated time until 60 min because these concentrations of PGE_2_ were demonstrated to induce potential *E*_max_ levels of cAMP formation on each receptor, as shown in Fig. 1A. As shown in Fig. 1B, in IBMX‐pretreated HEK‐EP2 cells or HEK‐EP4 cells, PGE_2_ was able to evoke nearly maximal activation after 15 min (EP2: 19.9 ± 4.10 pmol, EP4: 9.10 ± 2.33 pmol) of stimulation through both receptors in a similar manner (*E*_max_ at 60‐min stimulation, green line; EP2: 22.0 ± 3.38 pmol, orange line: EP4: 11.9 ± 2.18 pmol). However, without IBMX pretreatment, PGE_2_ was also able to evoke around 80% of the potential *E*_max_ level, peaking at around 15 min in HEK‐EP2 cells (lime line: apparent/pseude *E*_max_ (‐IBMX): 17.0 ± 6.72 pmol); then, the formed cAMP level slowly decreased 60 min after stimulation in HEK‐EP2 cells (6.10 ± 2.53 pmol). On the other hand, in HEK‐EP4 cells treated with PGE_2_, there was limited cAMP formation, peaking at approximately 15 min, without IBMX pretreatment (red line: apparent/pseudo *E*_max_ (‐IBMX): 0.609 ± 0.345 pmol). At 60 min after stimulation, the practically formed cAMP then decreased close to the basal level (0.344 ± 0.251 pmol).

This suggested that more than half of the potentially formed cAMP was degraded by the action of PDE under physiological conditions when EP2 receptors were treated with PGE_2_ for 60 min. However, close to 80% of the *E*_max_ level of cAMP accumulated when EP2 receptors were treated with PGE_2_ for 15 min, being an apparent/pseudo *E*_max_ (*E*_max_ (‐IBMX)). On the other hand, when EP4 receptors were treated with PGE_2_ under physiological conditions, the accumulated amount of cAMP was approximately one‐tenth, if any of the potential *E*_max_ level 15 min after stimulation. Thus, although the potential *E*_max_ level of PGE_2_‐stimulated cAMP formation in HEK‐EP4 cells was reported to be lower, approximately half than that in HEK‐EP2 cells, the practical amount of accumulated cAMP under physiological conditions in HEK‐EP4 cells may be less than one‐tenth that previously considered.

Of note, in HEK‐EP4 cells under the influence of IBMX, PGE_2_ treatment led to the formation of approximately half of the potential *E*_max_ level of cAMP in HEK‐EP2 cells, demonstrating EP4 receptor‐mediated adenylyl cyclase activity. However, without IBMX pretreatment, PGE_2_ accumulated markedly low levels of practically formed cAMP, suggesting that PGE_2_‐stimulated EP4 receptors exert greater effects on PDE activity, and/or PGE_2_‐stimulated EP2 receptors do not activate PDE to the extent of EP4 receptors, as similarly discussed for D‐type prostanoid receptors and EP2 receptors [14].

We previously reported that EP2 and EP4 receptors are able to activate at least three independent signaling pathways: cAMP‐mediated signaling, ERKs‐mediated signaling, and β‐catenin/TCF‐mediated signaling [11, 15]. However, as shown in Fig. 1, the cAMP‐mediated signaling of EP4 receptors may play limited roles in these pathways. Therefore, the comparative degree of participation in each signaling pathway by each receptor was evaluated. As shown in Fig. 1B, maximal cAMP accumulation was at 60 min with IBMX pretreatment in both HEK‐EP2 and HEK‐EP4 cells stimulated with PGE_2_. On the other hand, without IBMX, the maximum practical cAMP accumulation was at around 15 min, being an apparent/pseudo *E*_max_ (*E*_max_ (‐IBMX)). Thus, the practical amounts of cAMP formed without IBMX pretreatment can be regarded as partial agonist‐stimulated‐like results, whereas those with IBMX pretreatment are full agonist‐stimulated results. Using these amounts evoked by partial‐like and full agonist, the logical definition for the efficacy of each agonist in a system, as known as Tau values at 15 min and 60 min after PGE_2_ stimulation, was estimated by Black/Leff operational model calculation [12, 16, 17, 18, 19, 20]. The Black/Leff operational model can adapt the fitting of experimental results, e.g., *E*_max_ values and EC_50_ values, to the occurrence of ligand‐stimulated response cooperatively [12, 16, 17, 18, 19, 20]. Since the experimental concentration‐response curves may not reflect the stimulus‐response processes at all times, the Black/Leff operational model was utilized to determine the Tau values, the logical/operational efficacies [12, 16, 17, 18, 19, 20]; in this case, the Tau values of cAMP formation in the experimental conditions as shown in Fig. 1.

As mentioned above, the cAMP levels 60 min after PGE_2_ stimulation with IBMX pretreatment as shown in Fig. 1B may represent the potential maximal amounts of cAMP formed, which we previously examined. On the other hand, the cAMP levels at 15 min after PGE_2_ stimulation without IBMX pretreatment may represent the practical maximal amounts of cAMP formed in the system. As shown in Table 1, when cell lines were pretreated with IBMX, the fold difference between EP2 receptor‐stimulated cAMP formation and EP4 receptor‐stimulated cAMP formation was 1.85 at 60 min [EP2‐potential *E*_max_ (row 1): 22.0, EP4‐potential *E*_max_ (row 5): 11.9]. In contrast, without IBMX pretreatment, the fold difference between EP2 receptor‐stimulated practical cAMP formation and EP4 receptor‐stimulated practical cAMP formation was 27.9 at 15 min [EP2‐apparent/pseudopractical *E*_max_ (row 4): 17.0, EP4‐apparent/pseudopractical *E*_max_ (row 8): 0.610]. Therefore, the difference in receptor‐stimulated practical (and/or apparent) maximal amounts of cAMP accumulated between EP2 receptors and EP4 receptors may greater than 10 times that previously reported.

**Table 1:** The simulated affinity, *K*_A_ values, and logical definition for the efficacy, Tau values at 15 and 60 min after PGE_2_ stimulation.

	Row	time	IBMX	EC_50_ (nm)	pEC_50_	*E*_max_	*E*_max_ (%)	*K*_A_ (nm)	*pK*_A_	Tau
EP2 receptors	1	60	+	0.556	9.25	22.0	100	8.99	8.22	0.384
	2		−	4.33	8.36	[6.10]	28.0			
	3	15	+	0.556	9.25	[19.9]	100	29.7	7.53	5.86
	4		−	4.33	8.36	17.0	85.0			
EP4 receptors	5	60	+	0.0747	10.1	11.9	100	14.4	7.84	0.0298
	6		−	14.0	7.85	[0.344]	3.00			
	7	15	+	0.0747	10.1	[9.10]	100	15.0	7.82	0.0717
	8		−	14.0	7.85	0.610	7.00			

The *K*_A_ and Tau values at 15 and 60 min after PGE_2_ stimulation were estimated using the Black/Leff operational model. For calculation, the amounts of cAMP formed were calculated using the parameters obtained without IBMX pretreatment, which were regarded as partial‐agonist‐stimulated‐like results, whereas those with IBMX pretreatment were considered full‐agonist‐stimulated results.

Next, the Tau values, the logical definition of the efficacy of cAMP formation in a system, at 15 and 60 min after PGE_2_ stimulations were estimated by Black/Leff operational model calculation using the practical amounts of cAMP formed without IBMX pretreatment, which were regarded as partial agonist‐stimulated‐like results. As shown in Table 1, the Tau value of EP2 receptors at 60 min after stimulation was 0.384 (rows 1 and 2), whereas it was 0.0298 for EP4 receptors (rows 5 and 6). On the other hand, the Tau value of EP2 receptors at 15 min after stimulation was 5.86 (rows 3 and 4), whereas it was 0.0717 for EP4 receptors (rows 7 and 8). Simulations for EP2 and EP4 receptors at 15 min were performed using EC_50_ values that were obtained experimentally at 60 min as shown in Fig. 1. As previously described, the maximal practical amounts of cAMP formed may accumulate around 15 min after stimulation with PGE_2_; thus, the practical amounts of cAMP accumulated by the activation of EP4 receptors, 0.0717, were lower than those accumulated by the activation of EP2 receptors, 5.86, according to the Tau values.

As described in the introduction, EP2 and EP4 receptors were both initially identified as cAMP‐produced Gαs‐coupled receptors. EP4 receptors were then found that they have an additional signaling pathway involving the alliance of Gαi‐protein/phosphatidylinositol 3‐kinase (PI3K)/ERKs activation, which is absent in EP2 receptors [2, 6, 21]. Moreover, we also previously reported that both EP2 and EP4 receptors can similarly activate PGE_2_‐stimulated β‐catenin/TCF‐mediated signaling, which had the smallest/strongest potencies to maintain colorectal tissue homeostasis among the three independent signaling pathways, including the cAMP‐mediated pathway and ERKs‐mediated pathway [11]. For these calculations, the results of β‐catenin/TCF‐mediated signaling and ERKs‐mediated signaling, and previous cAMP‐mediated signaling were marked with asterisks in Table 2 and performed using the EC_50_ and *E*_max_ values obtained in this and previous studies [11].

**Table 2:** The estimation of log *R*, Δ log *R*, and ΔΔlog *R* from the experimentally measured parameters and operational model‐calculated parameters.

	row	pathway	EC_50_ (nm)	*E*_max_	*K*_A_ (nm)	Tau	log *R*	Δ log *R*	ΔΔ log *R*
EP2 receptors	1	cAMP 60 (potential)	0.556	22.0			9.26	−0.654	0.00
	2	cAMP 15 (potential)			29.7	5.86	8.30	−1.61	0.00
	3	cAMP*	0.548	23.2			9.25	−0.661	0.00
	4	ERKs*	12.6	7.65			7.90	−2.01	0.00
	5	β‐cat/TCF*	0.123	454			9.91	0.00	0.00
EP4 receptors	6	cAMP 60 (potential)	0.0747	11.9			10.1	−0.0700	0.584
	7	cAMP 15 (potential)			15.0	0.0717	6.68	−3.52	−1.91
	8	cAMP*	0.135	10.7			9.88	−0.325	0.336
	9	ERKs*	0.863	53.3			9.06	−1.14	0.873
	10	β‐cat/TCF*	0.0654	471			10.2	0.00	0.00

Each signaling pathway was compared including the cAMP results obtained at 15 min after PGE_2_ stimulation, which may be represented by the practical maximal amounts of cAMP formed in the system. Comparing signaling pathways, including cAMP amount obtained after 60 min of PGE_2_ stimulation, may represent the potential maximal amounts of cAMP formed that we previously examined. The logical definition for the efficacy, the Tau values of cAMP formation in a system, at 15 and 60 min after PGE_2_ stimulation, was estimated using the Black/Leff operational model. Using the operational model, the transduction coefficient value, log *R*, of each signaling pathway was then calculated to estimate Δlog *R*, the order of biased activity of each signaling pathway based on the β‐catenin/TCF‐mediated signaling pathway being 0.00. To further examine the ratio of biased activities among signaling pathways between EP2 and EP4 receptors, ΔΔlog *R* values were calculated based on the β‐catenin/TCF‐mediated signaling pathway and EP2 receptor‐mediated signaling being 0.00.

Thus, to use the operational model, the transduction coefficient value, log *R*, of each signaling pathway was calculated to estimate the Δlog *R* [18, 19], and the order of biased activity of each signaling pathway based on the β‐catenin/TCF‐mediated signaling pathway of each receptor being 0.00 because we previously demonstrated that this pathway is stimulated to a similar extent regardless of the EP2 or EP4 receptor subtypes [11]. As shown in Table 2, both EP2 and EP4 receptor‐mediated signaling pathways, the cAMP‐mediated pathway and ERKs‐mediated pathway, had negative values; therefore, β‐catenin/TCF signaling pathways have the most positive biased activities by both EP2 and EP4 receptors. In the case of the EP2 receptor‐mediated cAMP signaling pathway, 60‐min stimulation of PGE_2_ with IBMX pretreatment resulted in −0.654 [row 1: cAMP 60 (potential)], which was similar to the −0.661 obtained previously after 60‐min stimulation of PGE_2_ (row 3: cAMP*) [11]. Similarly, in the case of the EP4 receptor‐mediated cAMP signaling pathway, either 15‐ or 60‐min stimulation of PGE_2_ with IBMX pretreatment resulted in −0.0700 [row 6: cAMP 60 (potential)], which was close to the −0.325 obtained previously after 60‐min stimulation of PGE_2_ (row 8: cAMP*) [11]. By the EP2 receptor‐mediated cAMP signaling pathways, without IBMX pretreatment, the value obtained was −1.61 [row 2: cAMP 15 (practical)] after 15‐min stimulation of PGE_2_. Similarly, without IBMX pretreatment, the value obtained was −3.52 [row 7: cAMP 15 (practical)] after 15‐min stimulation of EP4 receptors with PGE_2_. This suggested that not only did the maximal practical amounts of the cAMP accumulate, but also that the biased activity of the signaling pathways evoked by both EP2 and EP4 receptors under physiological conditions is weaker than previously considered when compared with each β‐catenin/TCF‐mediated signaling pathway. Of particular interest, for EP2 receptors, the order of the biased activities is β‐catenin/TCF (0.00) > cAMP 15 (practical: −1.61) > ERKs (−2.01), whereas for EP4 receptors, it is β‐catenin/TCF (0.00) > ERKs (−1.14) > cAMP 15 (practical: −3.52).

Next, to further examine the ratio of biased activities among signaling pathways between EP2 and EP4 receptors, activity was quantified by calculating ΔΔlog *R* values [18, 19] based on the Δ log *R* values of EP2 receptor‐stimulated signaling being 0.00. As shown in Table 2, the ΔΔ log *R* value of ERK‐mediated signaling by EP4 receptors was 0.873 (row 9: ERKs*). Thus, EP4 receptors have positive biased activity of approximately 7.46‐fold greater for the ERKs‐mediated signaling pathway when compared with EP2 receptors. Similarly, the value for EP4 receptor‐stimulated cAMP‐mediated signaling pathways 60 min after IBMX pretreatment was 0.584 [row 6: cAMP 60 (potential)], similar to that obtained previously after 60‐min stimulation of PGE_2_, which was 0.336 (row 8: cAMP*). This suggested that with IBMX pretreatment, EP4 receptors also have positive biased activity for the cAMP‐mediated pathway when compared with EP2 receptors. However, without IBMX, the EP4 receptor‐mediated cAMP‐mediated signaling pathways had a negative value of −1.91 [row 7: cAMP 15 (practical)]. Thus, without IBMX pretreatment, in terms of the cAMP‐mediated signaling pathway, EP4 receptors have negative biased activity that is approximately 81.3‐fold weaker than that of EP2 receptors.

**Fig. 2:**
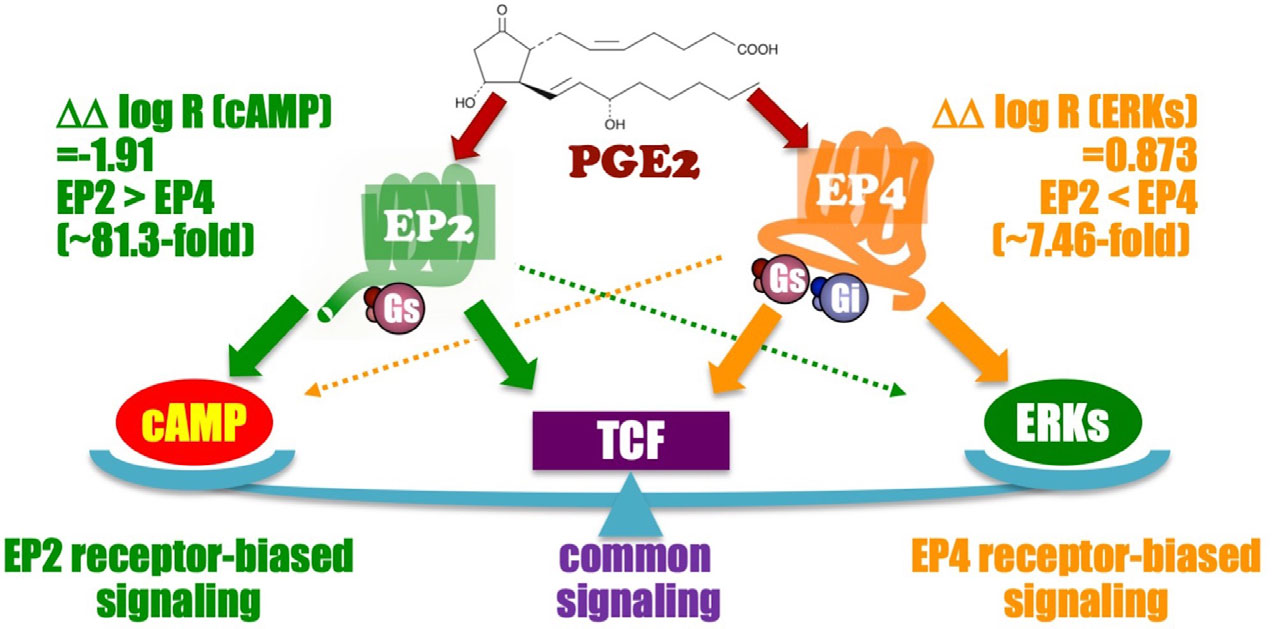
The schema shows that EP2 receptors have a biased ability to activate the Gαs‐protein/cAMP‐mediated pathway, whereas EP4 receptors have biased activity for the Gαi‐protein/ERKs‐mediated pathway. Along with β‐catenin/TCF‐mediated signaling, EP2 receptors have the ability to intrinsically activate the Gαs‐protein/cAMP‐mediated pathway, whereas EP4 receptors have strong biased activity for the Gαi‐protein/ERKs‐mediated pathway.

Taken together with β‐catenin/TCF‐mediated signaling, EP2 receptors have a biased ability to intrinsically activate the Gαs‐protein/cAMP‐mediated pathway, whereas EP4 receptors have strong biased activity for the Gαi‐protein/ERKs‐mediated pathway. Thus, EP2 and EP4 receptors may play different roles via the activation of distinct biased pathways, as depicted in Fig. 2.

As previously reported, Gαs‐protein/cAMP/PKA‐mediated signaling is widely recognized to regulate the inhibition of cellular growth [13], whereas Gαi‐protein/PI3K/ERKs‐mediated signaling is related to cancer malignancy [21, 22]. The proliferation and differentiation of normal colorectal epithelial cells were reported to be regulated by β‐catenin/TCF‐mediated signaling [23]; hence, this pathway is key in maintaining colorectal homeostasis. As such, homeostatic mechanisms may be tightly regulated by the balance of expression levels of EP2 and EP4 receptors, as we previously discussed [11]. Thus, when EP4 receptors are overexpressed, for example, by the reduction of butyrate in the environment as discussed previously [24], EP4 receptor‐mediated signaling can cause cancer malignancy signaling due to the unexpectedly lower formation of cAMP.

Of note, although EP4 receptors formed unexpectedly lower levels of practical cAMP under physiological conditions, these receptors activated PKA, albeit 1.5‐fold at higher most when compared with the vehicle‐treated control, as previously reported [10]. Thus, although little cAMP accumulated by EP4 receptor activation without IBMX pretreatment, EP4 receptors can activate significant Gαs‐protein‐mediated signaling; however, cAMP‐mediated signaling was much lower/weaker than previously expected and/or considered.

## Conclusions

EP2 and EP4 receptors have long been considered to share Gαs‐protein and cAMP‐mediated signaling to a certain extent. Although EP4 receptors have the potential to activate Gαs‐protein and produce cAMP, they may have little involvement in cAMP‐mediated signaling under physiological conditions. As cAMP‐mediated signaling has been regarded as playing a role in cell growth inhibition, EP4 receptors may not be able to evoke the growth inhibitory signaling, hence why EP4 receptors were reported to be closely related to the development of cancer malignancy. Although both receptor subtypes similarly activate the β‐catenin/TCF‐mediated pathways, as we reported previously [11], EP4 receptors primarily activate biased Gαi‐protein‐mediated pathways, whereas EP2 receptors may stimulate biased Gαs‐protein‐mediated pathway. Thus, after a certain period of time, EP2 and EP4 receptors may no longer be classified as equivalent Gαs‐coupled receptors but as substantially different receptors with distinct roles to maintain homeostasis in a coordinated manner.

## Conflict of interest

The authors declare no conflict of interest.

## Author contributions

KF, KS, and NK involved in formal analysis, validation, and methodology; KF involved in software; KF and HF involved in visualization and funding acquisition; JWR and HF involved in data curation and writing original draft; HF involved in investigation, conceptualization, supervision, project administration, and writing—review and editing.

## Data Availability

The data that support the findings of this study are available from the corresponding author, [HF], and KF upon reasonable request.
